# *In vitro* and *in vivo* anticancer effects of marmesin in U937 human leukemia cells are mediated via mitochondrial-mediated apoptosis, cell cycle arrest, and inhibition of cancer cell migration

**DOI:** 10.3892/or.2021.8065

**Published:** 2021-04-26

**Authors:** Lin Dong, Wen-Wei Xu, Hong Li, Ke-Hong Bi

Oncol Rep 39: 597-602, 2018; DOI: 10.3892/or.2017.6147

Following the publication of the above paper, a concerned reader drew to the Editor's attention that a pair of tumors in Fig. 10 appeared to have been duplicated, although one of the tumors appeared at a larger size in the figure relative to the first one. Furthermore, the flow cytometric plots shown in Fig. 2B in the above paper appeared to be remarkably similar to data presented in a paper published in *Phytomedicine* [Sui C-G, Meng F-D and Jiang Y-H: Antiproliferative activity of rosamultic acid is associated with induction of apoptosis, cell cycle arrest, inhibition of cell migration and caspase activation in human gastric cancer (SGC-7901) cells. Phyomedicine 22: 796–806, 2015].

After having conducted an independent investigation in the Editorial Office, the Editor of *Oncology Reports* has determined that the above paper should be retracted from the Journal on account of a lack of confidence concerning the originality and the authenticity of the data. The authors were asked for an explanation to account for these concerns, but the Editorial Office never received any reply. The Editor regrets any inconvenience that has been caused to the readership of the Journal.

